# Echo chambers and viral misinformation: Modeling fake news as complex contagion

**DOI:** 10.1371/journal.pone.0203958

**Published:** 2018-09-20

**Authors:** Petter Törnberg

**Affiliations:** Sociology Department, University of Amsterdam, Amsterdam, The Netherlands; University of Waterloo, CANADA

## Abstract

The viral spread of digital misinformation has become so severe that the World Economic Forum considers it among the main threats to human society. This spread have been suggested to be related to the similarly problematized phenomenon of “echo chambers”, but the causal nature of this relationship has proven difficult to disentangle due to the connected nature of social media, whose causality is characterized by complexity, non-linearity and emergence. This paper uses a network simulation model to study a possible relationship between echo chambers and the viral spread of misinformation. It finds an “echo chamber effect”: the presence of an *opinion* and *network* polarized cluster of nodes in a network contributes to the diffusion of complex contagions, and there is a synergetic effect between opinion and network polarization on the virality of misinformation. The echo chambers effect likely comes from that they form the initial bandwagon for diffusion. These findings have implication for the study of the media logic of new social media.

## Introduction

The way we become informed, debate, and form our opinions have changed profoundly with the advent of online media [1–5]. Today’s media is less organized through centralized decision-making, and more through complex cascade processes, where news items spread like wild-fire over social networks through direct connections between news producers and consumers—categories between which it is becoming increasingly hard to distinguish. Disintermediation has changed the way we as a society form narratives about our common world with promises of more egalitarian ways of meeting and discussing.

But despite early optimism about this ostensibly decentralized and democratic meeting-place, the online world seems less and less like a common “table” that “gathers us together” [6](p.52) to freely discuss and identify societal problems in a new type of “public sphere” [7]. Instead, it seems to bring forth the worst of human instincts: we cluster together into tribes that comfort us with reaffirmation and protect us from disagreement; “echo chambers” that reinforce existing perspective and foster confirmation biases [8–11]. Technology that was purported to help weave tighter the bonds between humans instead seem to has led to a fraying of our social fabric, as we are torn into social groups with separate world-views.

Simultaneous with this development is an on-going reduction in the quality and credibility of available information: while the Internet was initially hailed as an unprecedented source of easily accessible knowledge, it increasingly appears to instead have brought an information climate characterized by biased narratives, “fake news”, conspiracy theories, mistrust and paranoia. The digital world seems to provide fertile soil for the growth of misinformation, as studies show that false news diffuse faster, farther and deeper than true news in social networks [12]. This changing online climate is relevant not merely within the realm of social media, but may influence opinions and behavior also in other areas of human life [13–18]. This rapid cultural shift has quickly become an onerous threat, with viral misinformation now being seen as a major risk to human society [19].

There are certain signs that point to a link between these two phenomena—echo chambers and the spread of misinformation—since homogeneous clusters of users with a preference for self-confirmation seem to provide capable green-houses for the seedling of rumors and misinformation. A polarized digital space where users tend to promote their favorite narratives, form polarized groups and resist information that does not conform to their beliefs may be the fertilizer that makes the Internet so fertile for the growth of misinformation [20]. Armies of supporters for products, brands or presidents are quickly rallied, spreading their views in an environment where trust in traditional knowledge authorities is increasingly frail [21–25].

But the potential link between echo chambers and misinformation has proven difficult to evaluate due to the complexity underlying social media dynamics. The transition in information dynamics that digital media has brought can be usefully seen through the notion of *media logic*, i.e. the “particular institutionally structured features of a medium […] that will tend to structure particular perceptual and cognitive biases” [26] (p.63-64). This notion thus points to structural factors of a medium, which in the case of social media implies an entwined causal of distributed actors [27, 28]. Contemporary media logic has taken a distinctly postmodern turn with digital technology, as networks and identities now carry more weight than the truths of traditional authorities [29]. It has gone from the structured top-down logic of the machine—with centralized institutions and trained journalists making decisions from editorial chairs—to the decentralized and dynamic complexity of what resembles a swarm or herd—millions of dopamine-driven smartphone-users swiping through click-bait news on porcelain thrones [30, 31]. Such swarm logic often departs from the terrain accessible by human intuition, and travels into the realm of emergence and complexity where neither un-aided cognition or traditional scientific methods are able to disentangle the complex chains of causality [32].

This paper suggests an emergent causal link that could serve to connect the spread of misinformation and online echo chambers. The nature of this link is perhaps most clear through the lens of a metaphor: if we think of the viral spread of misinformation in a social network as akin to a wildfire [19], an echo chamber has the same effect as a dry pile of tinder in the forest; it provides the fuel for an initial small flame, that can spread to larger sticks, branches, trees, to finally engulf the forest (it should be noted that this metaphor has a specific and technical meaning: forest fires received significant early interest within complexity theory, see e.g. [33, 34], the metaphor also recalls Schelling’s [35] threshold model). Just as how segregation has been, famously, shown to involve emergent feedback processes [36], it is in this paper suggested that an emergent network effect results in that *misinformation and rumors spread easier in networks where there is a presence of an echo chamber*. The model implies that news originating in a segregated cluster of users tends to spread further than in a network without clusters. It furthermore shows that the mere coming together of users with similar views may be enough to increase the prevalence of misinformation, as virality increases with network homophily. It finally suggests that the combination between these two factors, arguably what defines an echo chambers, is synergetically more prone to induce global cascades than either taken in isolation. This could mean that echo chambers make fake news more viral, since information that resonates with biased clusters of users has a higher likelihood to spread through a network.

This paper thus uses a simple network model to study whether the network structure of echo chambers can in itself have an effect for the spread of information. Online echo chambers are modelled as set of users characterized by *opinion* and *network* polarization, i.e. they are clusters of like-minded users, that are (i) more separated from the rest of the network, and (ii) have a lower threshold for being convinced by a given narrative. This approach may provide direction for further study in online media dynamics in general, and the spread of misinformation in particular.

## 1 Modeling spread of misinformation on social media

The model presented here aims to be simple as this both increases generality and makes the results of the model easier to interpret and bring into a sociological narrative [37, 38]. Observing that there is a tendency for echo chambers to form around conspiracies and mistrust of traditional authorities, the question that this model asks is: if a diffusant is associated to an echo chamber, will this affect its virality in a social network? The model thus does not deal with the content of the diffusant, meaning that its dynamics applies to any type of information spreading from an echo chamber.

The model describes the spread of a news item on social media as a *complex diffusion in a social network*. This means that we understand a social network as a number of connected nodes—representing users—that each are assigned a threshold that describes how difficult they are to convince about a given narrative. If a large-enough fraction of their neighbors spread a given narrative, the node will be convinced, and will continue the spread of the diffusant. Hence, the threshold can be seen as representing to what extent a given news item or idea *resonates* with a certain user.

In this network, we understand an *echo-chamber* as a set of users characterized by two properties: *opinion* and *network* polarization. *Opinion* polarization means that they, in relation to a given question, are more inclined to share similar views. *Network* polarization means that they are more densely connected with each other than with the outside network. In other words, an echo chamber is a tightly connected set of nodes more inclined to share a common view on a given narrative. For the sake of simplicity, this model focuses on the existence of a single echo-chamber in a larger network.

This type of clusters in networks have generally been understood to constitute impediments to the spread of diffusion, as they reduce the number of the weak ties that have in turn been found to be central enablers of diffusion (see e.g. [39–43]). Modeling results have shown that things like information, diseases, and so on, have a harder time of spreading into and out of clusters, since these are less connected to the surrounding network, which means that they will also reduce the probability for network-wide cascades.

However, these results specifically concern diffusion of entities that require only single contact to spread. This makes sense in many cases, for example disease, but it has been argued to be a problematic assumption when studying behavioral phenomena (see e.g. [44, 45]). In these cases, *complex contagion*, is often a more plausible view: that multiple contact increases the likelihood for a contagion to spread. In these cases, weak ties have been shown to be less relevant, or even have a negative effect on diffusion, and there are situations where clusters may in fact be beneficial for the diffusion. But not always: even for complex contagions, clusters may have negative impact on diffusion. For example, Easley and Kleinberg [46] show using mathematical reasoning that given that cascades start outside of the cluster, that they are seen as successful only if every node in the network is activated, and that every node has the same number of neighbors, clusters will have negative effects for cascades.

We argue that online news and rumors are better characterized as a complex rather than simple contagions, since a news item or idea becomes more convincing with the number of people that argue for it [44]. For example, a single user in your network claiming that vaccines cause autism or that Russia is an important ally to the US may easily be disregarded as a nut-job. But if a large fraction of your social network argues for the same thing, it not only makes the argument seem more authoritative, but you may also feel the need to conform to your social group [47]. This majority effect becomes especially clear if we think of political discussions and online interaction not as much as a spread of information, but as having elements of group identity and belonging, an understanding that is becoming increasingly prevalent throughout the social sciences [48, 49]. Echo chambers are generally understood as also playing a role in the formation of interpretive frames and collective identities, rather than simply constituting a hub for information diffusion [50].

The second aspect of the echo chambers, *opinion* polarization, implying that their thresholds are lower than that of the surrounding network, is related to what in network terms is referred to as *homophily*: the probability that neighboring nodes have similar thresholds for activation. Moderate levels of homophily is generally understood to increase the network’s capacity for diffusion [51–54]. This can also be connected to the suggestion in critical mass theory that an initial group can solve the large group problem by creating a “bandwagon effect” [55–58]. This model differs from studies looking only at homophily (e.g. [51]) as it combines homophily with network clustering, to thereby capture the essence of a social network echo chamber.

The model here presented primarily differs from previous approaches in the literature by: (i) looking at the dynamics of complex rather than simple contagions, which may have important effects for the results; (ii) loosening the assumptions that *every* node in the network needs to be activated for a cascade to be understood as complete, and that each node has the same number of neighbors; (iii) exploring the systemic interaction between *network* and *opinion* polarization, i.e. between lower activation thresholds and level of clustering, as these are both characteristic of echo chambers. In other aspects, the model generally follows the established design of existing network cascade models that focus on how different factors affect the probability for activation to diffuse in a network [44, 51, 59].

### 1.1 Model design

We refer to the probability that activation will spread to a *majority of the network’s nodes*, given a certain distribution of network structures, as the “virality” of the diffusion. Since the connections between the nodes represent the existence of mutual social contact, the model uses undirected ties. The distribution of these ties follows the so-called *Erdős-Rényi* structure, where each tie is assigned with uniform probability, which is commonly used in this type of models [43]. The distribution of ties in an Erdős-Rényi network follows a Poisson distribution. To form the clusters, which here represent free social spaces, a fraction of the ties that span from inside the cluster to outside the cluster are removed and replaced with ties inside the clusters, resulting in more internal cluster ties than external. The fraction between internal and external ties represents the previously introduced notion of *network polarization*. For example, with a polarization value of 0.85, 85% of the cluster’s external connections are relocated, while a polarization value of 0 represents a standard Erdős-Rényi structure. This definition of network polarization captures the idea that echo chambers are clusters of more densely connected users.

A cascade is initiated by the activation of a randomly selected node and its neighbors. This could for example be in the form of an actor posting a blog post or tweet with misinformation. In each following time step, nodes that have more than a certain fraction—called their *threshold*—of their neighborhood activated themselves become activated—what has been referred to as a *complex contested contagion* [44]. This continues until a steady state is reached. If at this point, a majority of the network has become activated, the cascade is classified as successful.

Since the focus is on echo chambers, we assume that the cascade is initiated *inside* the cluster. Furthermore, we implement the previously introduced notion of *opinion polarization* as a parameter lowering the activation threshold for the cluster nodes relative to the average threshold, meaning that the nodes inside the cluster are more easily activated. This of course trivially has an effect of increasing the virality, and we hence compare to a control case, in which the reduction of threshold is assigned to random nodes in the entire network.

The primary question that we aim to investigate with this model is whether echo chambers have any effect on the virality of cascades, and to furthermore look at the interaction between opinion and network polarization in terms of virality.

### 1.2 Model implementation

We define: *P*_*n*_ as the network polarization parameter, *P*_*o*_ as opinion polarization parameter, *k* as the average degree of the nodes, *θ* is the activation threshold, *c* is the fraction of nodes belonging to the echo chamber. *E* is the total number of edges, *N* are number of nodes. For each combination of steps of parameter values, the model is run 1,000 times, with a new network structure generated for each run to compensate for network structure heterogeneity and allow for higher generality and robustness. Parameters *P*_*o*_, *P*_*n*_, and *θ* are systematically varied over an interval, to find how these parameters affect the model (additionally, other parameters where tested for sensitivity in separate runs.) With 100 steps in *P*_*o*_ and 200 steps in *θ* and 12 steps in *P*_*n*_, the model is run a total of 2.4 * 10^8^ times, which is arguably a reasonably thorough exploration of parameter space, thereby allowing an in-depth assessment of the model dynamics. Furthermore, these runs were evaluated for a number of network sizes, to further validate their robustness and relevance.

In each such run, an *Erdős-Rényi* network structure is constructed in the following way. *N* nodes are created, *cN* of which are specified as belonging to the echo chamber. The specified mean degree *k* is divided by two (since the network is undirected and any edge has two sides) and multiplied with the number of node edges, i.e. $E=N\frac{k}{2}$. From this set, *P*_*n*_ *kE* edges are selected where exactly one of the connected nodes belong to the cluster. These are removed, and replaced by edges where both nodes belong to the cluster. Following this, nodes outside the cluster are set to have activation threshold *θ*, and nodes in the cluster are set to have threshold *θ* − *P*_*o*_. A random node in the cluster is then selected, and set as activated, to act as activation seed. All nodes connected to this node are also set as activated. After this, the following is repeated until no more changes occur: for each node, if the fraction of activated connected nodes is larger than the node threshold, the node is set as active in the next step. If the fraction of active nodes at the end of such a run is larger than 0.5, the cascade is considered global (this definition prevents the mere activation of the echo chamber to affect the measured level of virality, and follows previous research, see e.g. [45]). This procedure is repeated 1,000 times with new random network structure for each parameter step. *Virality*, *V*(*θ*, *P*_*n*_, *P*_*o*_), is defined as the fraction of times in these runs that a majority of the nodes are activated.

## 2 Results and analysis

We will now analyze the results of the model through a step-by-step process illustrating and describing the model output through different graphs.

### 2.1 Relation between network polarization (*P*_*n*_) and virality (*V*)

We start by looking at how the presence of a network polarized cluster affects the virality—i.e. the likelihood that the complex contagion diffuses to a majority of the network nodes—without taking opinion polarization into account.

**Fig 1:**
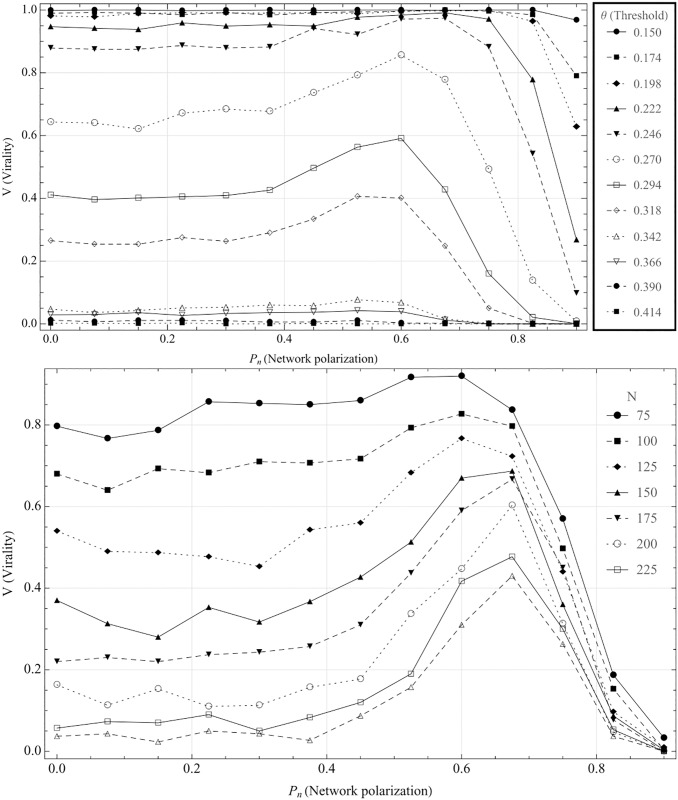
Virality as a function of network polarization. For parameters, see Table 1. This figure shows the effects of the echo chamber with *P*_*o*_ = 0, i.e. without opinion polarization (implying no difference in activation threshold between the cluster and the overall network.) The results are shown for different average threshold levels. As can be seen, the cluster increases the virality until the network polarization passes 0.6, from which it starts having a negative impact on virality. The lower graph shows the effects of varying the number of nodes in the network. The lower graph shows the variance for network polarization with *θ* = 0.27 for varying node counts, to show that the results are robust for varying network sizes. As can be seen, the virality falls with larger network sizes, however, the effects of having a cluster present seems to possibly increase with network size. The lower graph was averaged over 300 iterations, with degree 8, and 20% of nodes in cluster. (Runs performed with opinion polarization showed the same result).

Looking at Fig 1, it is clear that the presence of a cluster *has a positive effect on virality*. For example, at *θ* = 0.270, a completely random network without cluster has an around 65% chance of a global cascade, while for $P_{n}=P_{n}^{c}$ (i.e. at the optimal level of network polarization with regard to virality) it is more than 85%. This is intuitively rather unexpected, as it implies that the network polarization itself—rather than only together with opinion polarization—impacts through emergent structural effects. This implies that it is not necessary for the individuals associated to echo chambers to be unusually inclined to believe a narrative for them to have disproportionate effect on its diffusion: *the simple fact that the diffusion originates in an isolated cluster is enough to increase virality*. It should also be noted that this is yet another difference in the cascade dynamic between complex and simple contagions [44].

**Table 1:** Model parameters.

Parameter	Value
*N* (network size)	100
*k* (average degree)	8
*c* (cluster fraction)	0.2
Δ*P*_*o*_ (step size for opinion polarization)	0.03
Δ*P*_*n*_ (step size for network polarization)	0.075
Δ*θ* (step size for threshold)	0.0015
Iterations for each parameter set	1,000

These parameter values were used to generate all graphs, unless otherwise specified in the figure text. The step sizes constitute the “resolution” of the parameter values. The *iteration count* describes how many times the model was run for each parameter step, with new random network structures for each run to compensate for network heterogeneity.

We can see in Fig 1, that the effects of having a cluster increases gradually from around *P*_*n*_ = 0.4, and peaks at *P*_*n*_ = 0.6. At *P*_*n*_ > 0.7, virality falls quickly, likely as it becomes increasingly difficult for the cascades to spread from the cluster as the number of external connections are reduced.

As can be seen in the figure, by comparing the slopes of the different lines, the effect of having a cluster depends strongly on the threshold level: for threshold levels where no cascade is possible, or where cascades are almost certain to occur, the presence of clusters of course has little effect. The threshold levels that are most interesting are thus the ones where the effect of the presence of the echo chamber is as large as possible, i.e. the *θ*_*c*_ for which $V\left(\theta_{c},P_{n}^{c},P_{o}\right)-V\left(\theta_{c},0,P_{o}\right)$ (henceforth denoted $\Delta V(\theta_{c},P_{n}^{c},P_{o})$) is maximized, for some given *P*_*o*_ and where $P_{n}^{c}$ is, similarly, the network polarization level for which virality peaks. In the following analysis, we will thus focus on these threshold levels, which we refer to as the “critical threshold”, or *θ*_*c*_. As can be seen in Fig 1, the effect of varying *θ* is far from linear and, unsurprisingly, has a large impact on virality. To further investigate the effects of changing threshold levels, we begin by looking at how varying the average threshold affects virality for different parameter settings.

### 2.2 Relation between virality (*V*) and threshold (*θ*)

We now also introduce *opinion* polarization, *P*_*o*_, in the echo chambers, i.e. the threshold is reduced inside the cluster compared to the outside. We begin by looking at how the average threshold level, *θ*, affects virality. This sheds light on the question of how the quality or resonance of a certain news item or idea affects its possibility to spread in the network, which in turn constitutes a central aspect of the media logic of social media. Is the spread of an idea directly proportional to the resonance of that idea in the network, as would be naïvely expected?

**Fig 2:**
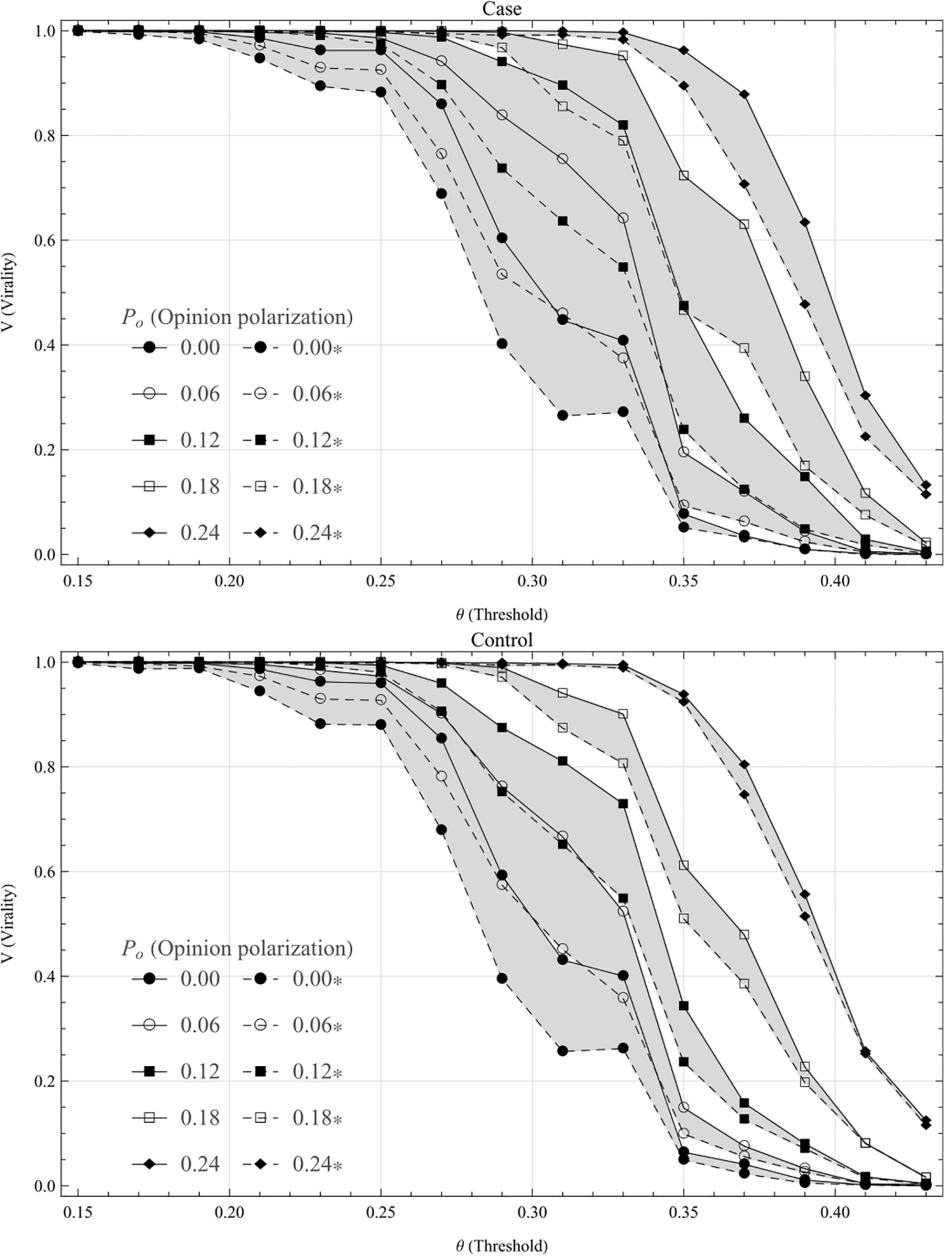
Virality as a function of threshold. Shows virality (*V*) as a function of the average threshold value (*θ*), for different levels of opinion polarization (*P*_*o*_). The upper, filled line for each *P*_*o*_ shows virality at the critical level of network polarization (i.e the *P*_*n*_ for which the effects of the presence of a cluster, *V*(*θ*_*c*_, *P*_*n*_, *P*_*o*_) − *V*(*θ*_*c*_, *P*_*n*_, 0) = Δ*V*(*θ*_*c*_, *P*_*n*_, *P*_*o*_), peaks for some given *P*_*o*_, denoted $P_{n}^{c}$) and the lower, dashed line for an Erdős-Rényi network. The upper figure shows the case (the nodes in the echo chamber are opinion polarized), while the lower shows the control (opinion polarization is assigned to random nodes in the network.) Two things should be noted: (i) The relationship between threshold and virality is far from linear, and (ii) as can be seen, the distance between the dashed and filled lines becomes significantly wider in the case than in the control: *this tentatively suggests the presence of a synergic interaction between network and opinion polarization*.

As can be seen in Fig 2, the relationship between threshold (*θ*) and virality (*V*) is far from linear: the transition in virality is fairly rapid. The slope seems to be equally steep for the different settings, the difference between which seem to be mainly expressed in an offsetting of the transition to lower threshold values. The graph thus shows how relatively small changes in threshold can result in increased probability for rapid global cascades. In other words, the virality of a narrative is not proportionally related to its quality or resonance, but small differences can have big effects. This illustrates an important aspect of the logic of new social media.

It is no surprise that opinion polarized nodes result in higher virality, in both the case and the control, since the definition of opinion polarization here is that some nodes have a lower threshold, which naturally increases the probability for transitions. More unexpected and interesting are the differences between the case and the control. For example, the surface between the dashes and filled line are larger with opinion polarized echo chambers than with the control, in particular for higher levels of opinion polarization (*P*_*o*_). This implies that *the effects of having an echo chamber is larger when the echo chamber is also opinion polarized*. This in turn indicates that there is a synergetic effect between opinion polarization and network polarization.

To study this closer, we return to looking at virality as a function of the level of network polarization (as in Fig 1), but this time, we include also opinion polarization, *P*_*o*_.

### 2.3 Relation between virality (*V*), and network (*P*_*n*_) and opinion polarization (*P*_*o*_)

**Fig 3:**
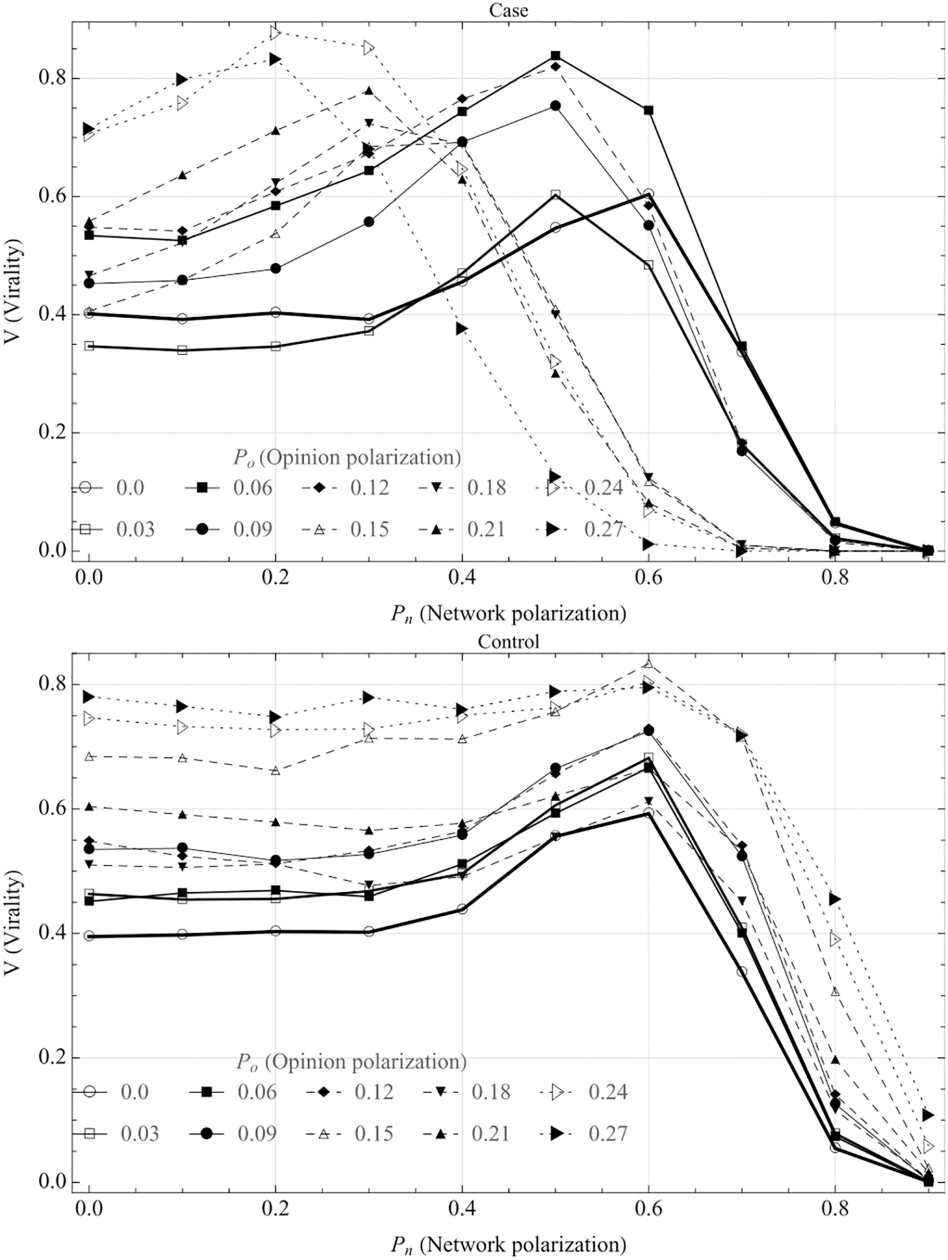
Virality and opinion polarization. In these graphs, we look *V*(*θ*_*c*_, *P*_*n*_, *P*_*o*_) (i.e. at the critical threshold level) for each level of *P*_*o*_ (opinion polarization) (note: since *θ*_*c*_ varies with *P*_*o*_, the lines in the graphs cannot be compared with regard to the absolute level of virality.) The different lines denote different level of opinion polarization (*P*_*o*_). The upper graph shows the case (when the echo chamber is polarized), and the lower the control (when opinion polarization is assigned to random nodes in the network.) As can be seen, in the case, *P*_*o*_ (the level of opinion polarization) affects $P_{n}^{c}$, i.e. at what level of network polarization that virality peaks. In the control case, there is no such effect.

Fig 3 shows the relationship between network polarization, opinion polarization and virality. Comparing the case and the control, there are multiple striking differences. First, the effect of having an echo chamber (i.e. $\Delta V(\theta_{c},P_{n}^{c},P_{o})$) is significantly larger for the case than the control. Second, in the case, $P_{n}^{c}$ (i.e. the level of network polarization for which virality peaks) depends on *P*_*o*_ (opinion polarization), but in the control, it remains constant. Looking at how $P_{n}^{c}$ changes as a function of *P*_*o*_, we see that the peak level gradually shifts to the left with increasing opinion polarization, i.e. toward lower levels of network polarization (note that the lines cannot be compared with regard to the level of virality, since *θ*_*c*_ varies with *P*_*o*_.) This suggests that *with higher levels of opinion polarization, virality peaks at lower levels of network polarization*. In other words, this may imply that if an echo chamber is highly coherent in terms of views on a specific news item or idea, its effects on virality becomes larger if it is more well-connected with the surrounding network.

**Fig 4:**
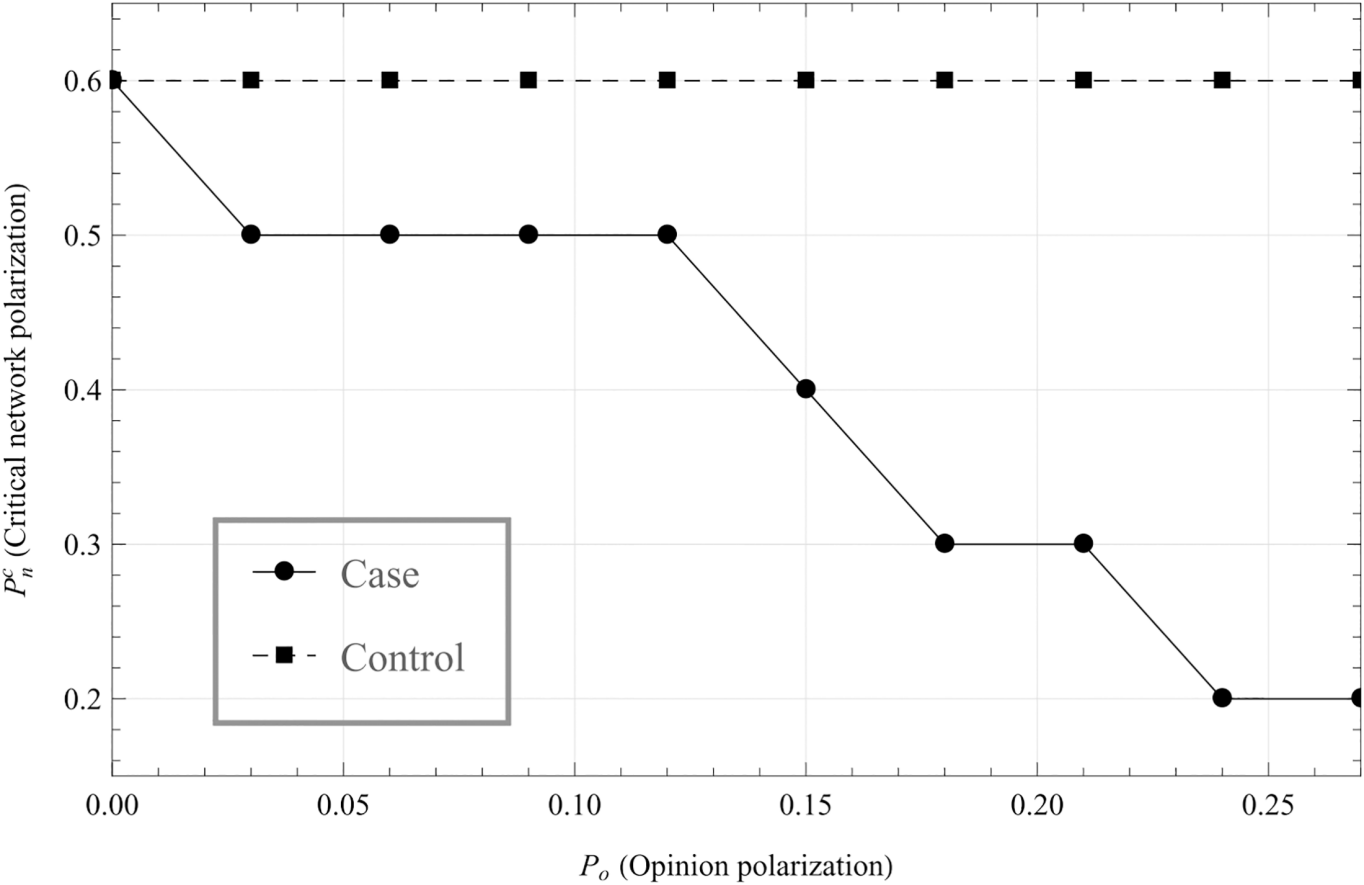
Critical network polarization as a function of opinion polarization. This graph shows how $P_{n}^{c}$ (the network polarization for which the highest virality is reached) depends on *P*_*o*_ (the opinion polarization of the cluster). Comparing to Fig 3, this shows the network and opinion polarization at each point at which virality peaks. As the graph shows, the optimal echo chamber has more external connections and fewer internal connections as *P*_*o*_ increases. In the control (dashed line), there is no interaction effect between network and opinion polarization.

We can look more closely at this relationship by plotting the network and opinion polarization at each point which virality peaks, giving optimal network polarization as a function of the level of opinion polarization. This is shown in Fig 4.

Fig 4 indicates that echo chambers with higher levels of opinion polarization have the most effect on virality when they have more external connections (i.e. lower *P*_*n*_). A plausible explanation for this is that it follows from the levelling between having enough internal ties to enable cohesion of the cluster, but having as many external connections as possible in order to spread the cascade globally. Since opinion polarized echo chambers need fewer internal ties to achieve internal cohesion, this implies that these ties have more impact when external.

This suggests that what is playing out in the dynamics of diffusion from echo chambers follows along the lines of Ghasemiesfeh et al. [60], who observe that one can distinguish two phases in the diffusion process: first, the cascade spreads locally via strong, short-range ties, and gathers the critical momentum it needs to transition into the second phase. In this second phase, the diffusion starts to spread also through long-range ties, to the rest of the network. Hence, in the first phase, strong local connections are beneficial, while the second phase is more similar to simple contagions, spreading quickly through weak ties.

**Fig 5:**
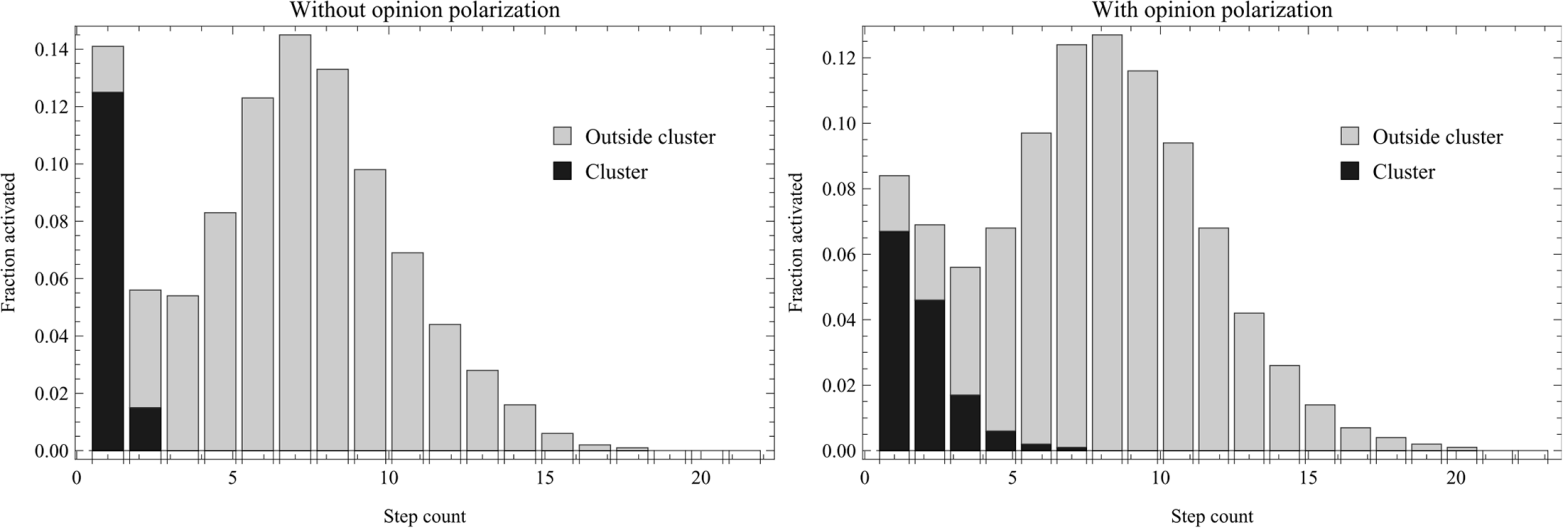
Order of node activation. Illustrates the order in which the node activation occurs in successful cascades, by showing the fraction of echo chamber and non-echo chamber nodes to be activated at each time step. The left graph has no difference in activation threshold between inside and outside the cluster, while the right has *P*_*o*_ = 0.2. As can be seen, the cluster is activated the first few steps, while the bulk of the non-cluster nodes are activated later, peaking around step 6 or 7. The runs are averaged over 1000 iterations, *P*_*n*_ = {0.7, 0.75, 0.85}, with *θ* = 0.27.

This suggests the hypothesis that echo chambers are beneficial for virality as they provide a boost to the first phase, by growing an initial momentum: echo chambers provide a foundation from which the global cascade can then follow. If this is indeed the way the role that echo chambers play in diffusion processes, successful viral spread should take the form of an initial wave of activation inside the echo chamber, followed by a second longer wave of activation in the larger network. To explore whether this is the case, we can look at the order in which nodes in the network are activated in successful spread. This is shown in Fig 5. As can be seen, there is indeed two phases of activation: first, the activated nodes almost exclusively belong to the echo chambers, second, it spreads to the larger network. This implies that a such a two-phase process is indeed playing out, and that the echo chambers may contribute to it.

So, we have thus far noted that there is an interaction effect between network and opinion polarization in echo chambers, and that both have a positive effect on virality. However, the perhaps most pertinent question remains: are there any synergic effects between these two factors with relation to virality? In other words, is the effect on virality of an echo chamber greater than sum of its parts? To explore this, we look at the effect of the echo chamber on virality as a function of opinion polarization.

### 2.4 Virality (*V*) as a function of opinion polarization (*P*_*o*_)

**Fig 6:**
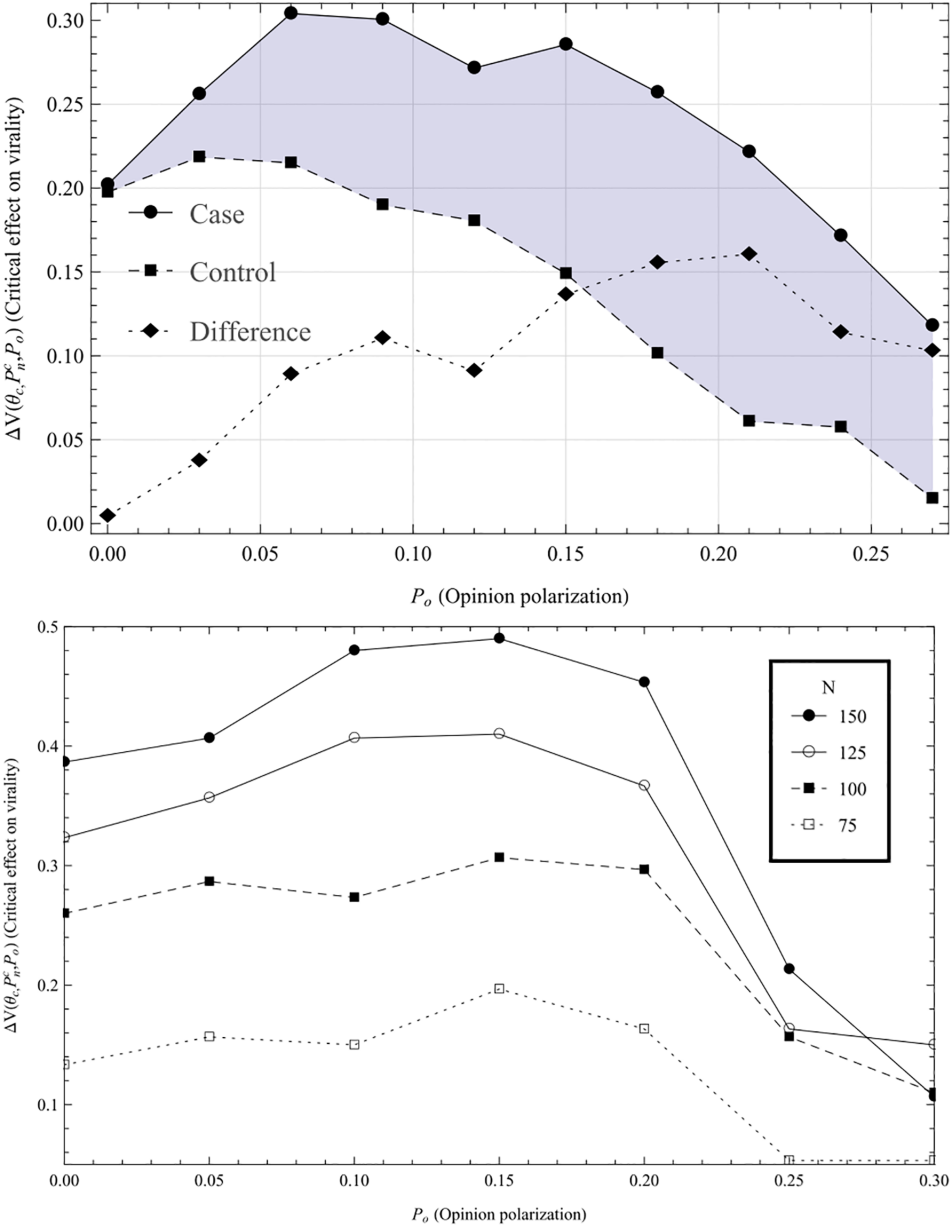
Effect of echo chamber. The graph shows the effect of the presence of the echo chamber for the network polarization level with highest effect, as a function of opinion polarization (i.e. $\Delta V(\theta_{c},P_{n}^{c},P_{o})$ as a function of *P*_*o*_). In other words, looking at Fig 3, for each level of opinion polarization, it substracts the virality without an echo chamber from peak virality, $V\left(\theta_{c},P_{n}^{c},P_{o}\right)-V\left(\theta_{c},0,P_{o}\right)$. The dashed line represents the control case where the opinion polarization has been randomly assigned. As can be seen, the presence of an opinion polarized echo chamber has more impact than the presence of one that is not opinion polarized. In the control case, there is little positive interaction between network and opinion polarization. The lower graph shows the same curve for different network sizes, to show that the results are robust for varying network sizes. As can be seen, the effects of having a cluster present actually increases with the network size. Lower graph runs were averaged over 300 iterations, with average degree 8, and 20% of nodes were assigned to be part of echo chamber.

Fig 6 shows the effect of an echo chamber with an optimal level of network polarization as a function of opinion polarization. In other words, the graph shows the impact of the presence of an echo chamber depends on how opinion polarized the echo chamber is. What we can see from the graph is that the effect of having an echo chamber increases with higher levels of opinion polarization, up until a level where the effect pans out, and then starts to decrease. Another way to put this is that up until a certain level of opinion polarization, the more opinion polarized the echo chamber is, the more effect does its presence have on virality. In the control case, there is almost no additional positive effect on virality from increasing opinion polarization. Indeed, when the opinion polarization is high, the presence of a cluster makes virtually no difference.

This implies that we indeed do have a synergistic relationship between opinion and network polarization for network virality. That this effect becomes negative at high levels of opinion polarization may be explained by Fig 4; as opinion polarization increases, lower level of network polarization is needed to maintain internal coherence in the echo chamber. So, with high opinion polarization, the optimal network polarization becomes lower. This also implies that the difference between having a cluster and not becomes smaller, which in turn means that not having a network polarized cluster becomes more efficient (this can most clearly be seen in Fig 3.)

Hence, we have two ostensibly contradictory results, as the effect of the cluster increases with higher opinion polarization, but the more opinion polarized the cluster is, the less network polarized should it be to maximize virality. The latter effect counteracts the former, and as the opinion polarization increases, this counteraction becomes stronger until the difference between having cluster and not becomes nominal.

In summary, we can hence conclude that the model implies that the existence of an echo chamber increases the virality in networks, and that the reason for this is that the echo chamber constitutes a “protected space” in which an idea or narrative can find a firm footing for further diffusion through the network. This suggests a theoretical connection to the “innovation niches” of socio-technical transitions, which are argued to act as “safe havens” in which new technologies can develop, free from market pressures, before spreading to the general market (see e.g. [61]). It also relates to the observation that while long ties are highly beneficial for the spread of simple contagions, complex contagions first have to spread locally before they can take advantage of such long-range ties. This also accounts for the synergetic effect between opinion and network polarization, as, if the echo chambers nodes have a very low activation threshold, they are likely to be activated even without being strongly separated from the rest of the network, meaning that highly opinion polarized groups have increased opportunity to focus on weak external ties.

### 2.5 Echo chambers in empirical networks

While these dynamics play out on the generated Erdős-Rényi networks, real world social networks differ in many ways from the type of simple networks that are generally used in this type of models. For instance, empirical social networks, in particular online variants, are well-known to display highly uneven degree distributions, high local clustering coefficient, and having multiple clusters or echo chambers. Thus, to validate that these observed dynamics are indeed playing out also in real-world networks structures, simulations were run also on empirical data. While using empirical data strengthens the claim that these emergent dynamics are in fact playing out also in real world social media, it does however also make it more difficult to separate the causal mechanism at play, as the networks structures will differ in a multitude of ways. Because of this, empirical and generated networks have somewhat different purposes, both being invaluable to investigate the phenomenon at hand.

To acquire empirical social networks for this analysis, the data presented in [62] were used. This dataset is gathered from Twitter, containing all retweet between politicians in a number of countries during 6 months (for details and definitions, see [62].) As the authors of the dataset argue, retweets are used by politicians to show their allegiances, therefore providing a link to an underlying network structure. These networks have central nodes—often party leaders—and are clustered along party lines, as party members to varying degrees tend to retweet within their party. The data are especially appropriate for this simulation as they are of relatively manageable size, thus allowing a thorough exploration of parameter space, they have a ground truth in the form of the political parties of the nodes, and they have a natural boundary following from this ground truth, meaning that it is not necessary to select the nodes to include on the basis of some arbitrary criteria.

To identify the effects of network polarization, a large number of networks structures are needed. Networks are generated from this data by seeing retweeting as a stochastic process revealing an underlying social network. The number of tweets between nodes is thus used to set the probability for a tie to exist between two nodes, which is then used as a set of probabilities from which a given number of edges are drawn. This allows us to generate networks with varied average degree by, in a sense, simulating varying the time interval during which the data were collected. Since data is collected over a finite time interval, meaning that retweets between all nodes will not yet have been collected, a low base probability *ϵ* is added to each network edge (here, *ϵ* = 0.1) to allow us to generate a network with higher average degree. This stochastic approach allows creating a large number of networks that describe the network properties of the data, while reducing the influence of the average network degree on the model outcome.

To additionally reduce the impact of networks differences, networks and parties were filtered on their size. The countries with between 80 and 150 nodes, and their parties of between 5 and 40 nodes were selected, generating 200 random networks for each of the 37 combinations. This resulted in a total of 7,400 generated networks. For each such network, opinion polarization (*P*_*o*_) and threshold (Θ) were varied, running 5 iterations for each combination, resulting in a total of 18.5 million model runs. An average degree of 8 was used in these runs. The level of network polarization of the clusters were calculated on these networks, using the same procedure as in previous simulations, and rounded off to closest 0.05.

**Fig 7:**
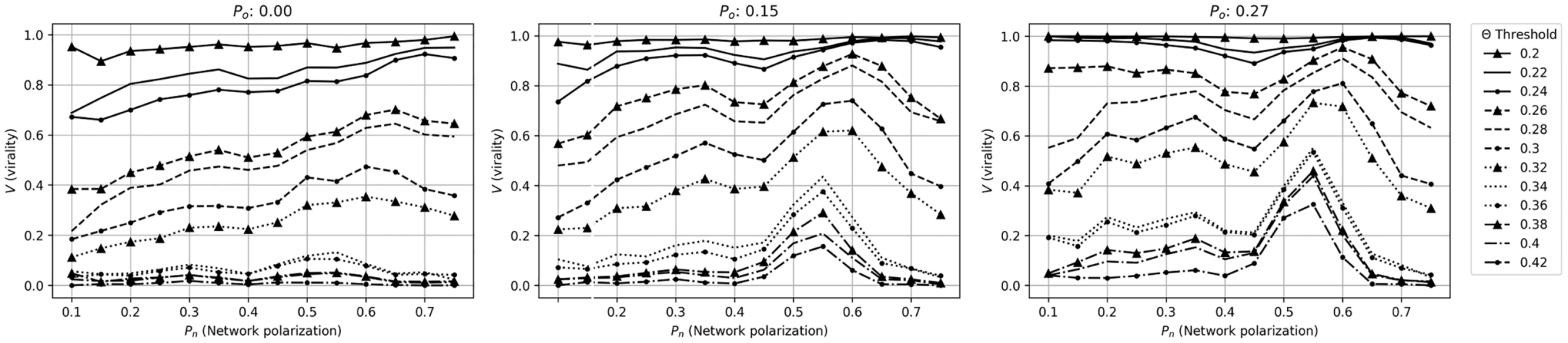
Virality as a function of network polarization in empirical networks. These figures correspond to Fig 1, and show the effects of network polarization on virality for different activation thresholds. As can be seen, virality (*V*) is affected by network and opinion polarization. As opinion polarization (*P*_*o*_) increases, the effect of network polarization (*P*_*n*_) becomes stronger, and we see a familiar peak at around *P*_*n*_ = 0.55 to 0.6. For higher values of network polarization, virality starts to fall.

Fig 7 show the result of these simulations. We see that the familiar dynamics are at play also in these empirical networks: virality increases with network polarization, and as opinion polarization increases, the effect of network polarization on virality becomes significantly stronger.

**Fig 8:**
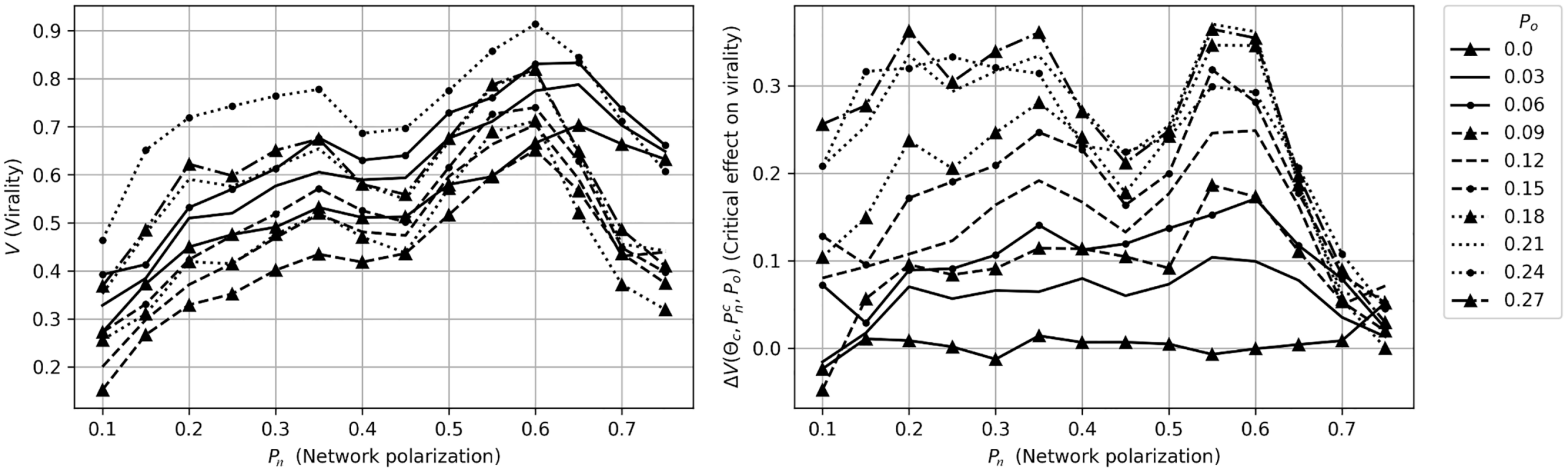
Critical virality on empirical networks. The left figure corresponds to Fig 4, showing the data from Fig 7 at critical threshold, i.e. the threshold where the impact of the cluster is the highest. (As in Fig 4, comparison between the lines should be done with care, as they represent different values of Θ.) The right figure shows the difference between the case and the control for these critical threshold levels. The figure shows that there is indeed a strong interaction effect between political and network polarization, also when the model is run on empirical networks.

Looking at the critical activation threshold for each opinion polarization level in Fig 8, the pattern observed in Fig 7 becomes even clearer: the cluster has a strong effect on virality, in particular for higher values of *P*_*o*_.

While these results overall match with the results on the Erdős-Rényi networks, some interesting differences can be noted. One such difference to the model dynamics on generated networks is that the virality peak does not vary as clearly over *P*_*n*_ as *P*_*o*_ changes (compare to Fig 4). This difference is likely the result of that while the generated networks had to, in a sense, prioritize between internal or external ties, these empirical networks are not restricted in this way, as they can vary their total number of connections. In other words, different clusters can vary in their internal and external connectivity, and it is quite possible for a party to have plenty of both internal and external ties. In fact, these are rather likely to correlate, as parties with highly active users will tend to tweet more both internally and externally.

The right figure in Fig 8 shows the difference in activation threshold at the critical level between the case and the control run. As the figure illustrates, higher levels of opinion polarization result in the echo chamber effect becoming stronger. However, there is little to no effect when political polarization is 0—despite that the left figure shows a significant effect of network polarization also without political polarization. This ostensibly unexpected result is in fact not very surprising: since these networks will tend to consist of multiple clusters, randomly activating a node—as in the control case—will still tend to activate a node within a cluster. When political polarization increases, however, the echo chamber effect comes into effect, and the difference between case and control grows significantly.

In summary, these empirical runs show that the observed dynamics are indeed at play also in empirical networks, with multiple clusters and uneven degree distribution. Despite that the level of network polarization co-varies with various related structural differences, there is a clear correlation between virality and network polarization, influenced by political polarization. This strengthens the claim that the model dynamics are at play also in real-world social media.

## 3 Conclusion

The model presented in this paper indicates that there may be general structural effects of echo chambers that contribute to the viral spread of misinformation. The model adds to the existing literature on network diffusion (see e.g. [44, 51, 59]) by showing that a combination between clustering and homophily has disproportionate effects on the capacity for complex diffusion, as it contributes to initiating a band-wagon effect. This stands in sharp contrast to both intuition and existing research on the spread of simple contagions, where clusters have instead been found to reduce virality [43]. This link between the presence of a polarized cluster and the spread of a complex contagion indicates a possible connection between echo chambers and the spread of misinformation. In other words, the result of this model suggests that echo chambers may be linked to the spread of misinformation through an emergent network effect. When misinformation resonates with the views of an echo chamber, the chamber can function as an initial platform from which the diffusion can occur globally through weak ties.

The model furthermore suggested that the combination between *opinion* and *network* polarization, quintessential of echo chambers, results in a synergetic effect that increases the virality of narratives that resonate with the echo chamber. This means that the simple clustering together of users with a deviant world-view is enough to affect the virality of information items that resonate with their perspective.

More broadly, this suggests that not only algorithmic “filter bubbles” [63] affect what news and perspectives we are exposed to online, but that the mere fact of social media permitting a dynamic of social clustering can change the dynamics of online virality. The possibility of self-segregation [64] can therefore affect not only what the segregated users see, but also what perspectives non-segregated users are exposed to. This can occur as subtle and complex network dynamics of the interaction structure of social media can play into the diffusion dynamics, in ways that are not necessarily even understood by the developers of the media platforms.

While simulations offer an unparalleled possibility to study specific causal mechanisms, it should however be noted that the result of a computational model is not enough to draw definitive conclusions about real world dynamics, since the observed mechanism may be overshadowed by other factors. The simplicity of the model described in this paper, while having the benefit of providing generality and permitting a thorough exploration of parameter space, also means that there are many factors that are not taken into account in the model. Additional research is therefore necessary to investigate whether the described mechanism is in fact in play in real world social media.

By engaging in this exploration, this paper constitutes a step toward the study of the “media logic” [27] of social media, which, as has been noted by multiple scholars, differ in important ways from the more institutionalized logic of traditional media [26, 28]. Since social media logic is the result of complex cascades and emergent effects, they require the use of computational models to study and discern their often-surprising dynamics [32]. Although clearly of central relevance for the understanding of the contemporary media landscape, these questions remain under-researched and poorly understood among both scholars and the lay public.

A fascinating self-similarity can be noted here, as the type of unintuitive complex dynamics characteristic of social media has been observed to be precisely what misinformation and conspiracy theories tend to seize upon: scholars have noted that conspiracy theorists often construct narratives that attempt to “restore a sense of agency, causality and responsibility” [65] (p.1). This seems related to a type of modernist thinking of society—what Andersson and Törnberg [38] refer to as the “complicated” system perspective—which would follow from approaching social media through the lens of traditional media. In this way, conspiracism is not only a symptom of the breakdown of knowledge authority, but of the breakdown of intuitive causality itself, as society becomes an increasingly complex system [66, 67]. In line with this, Gambetta and Hertog [68] show that engineers are over-represented among extremists, and argue that this comes from seeking the order and hierarchy of machines also in the social world. Conspiracy theories hence tend to describe a society under the conscious control of a global elite, seeking order and agency in a world which is becoming more and more like a leaf in the wind of social processes that are emergent and intangible, contingent and plural.

This is part of the larger question of how social media has affected our social and political processes. While online platforms have been thought of as equalizers and social levelers, which through disintermediation could provide equal opportunities for all, they in practice seem rather to result in the perpetuation and reinforcement of processes of stratification and inequality, as well as in self-segregation and tribalization [69]. The disappearance of media intermediation seems not to have, as was believed, fostered a space for direct meetings in a sort of online Habermasian public sphere, but rather to have implied that the “world between them has lost its power to gather them together, to relate and to separate them” [6] (p.52). As we find ourselves in this new condition of “worldlessness”, we go in search of new communities, new worlds in which we may again feel a sense of togetherness. These communities become places “where we see all people suddenly behave as though they were members of one family, each multiplying and prolonging the perspective of his neighbor. […] The end of the common world has come when it is seen only under one aspect and is permitted to present itself in only one perspective.” [6] (p.57-58).

The question of how to resolve the issues of misinformation and polarization, now considered among the main threats to human society, hence concerns how to design social media technology as to again constitute the foundation for a common world; how to shape our digital spaces as to help weave, rather than fray, our social fabric. For this, studies of the emergent externalities of the interaction structures of social networks will likely need to play a central role.
